# An Historical and Ætiological Essay on the Influenza

**Published:** 1839-01

**Authors:** 


### ART. IV.

1. *Die Influenza. Ein historischer und ätiologischer Versuch.* Von HEINRICH SCHWEICH, Dr. der Med. und Chir.—*Berlin*, 1836.

*An historical and ætiological Essay on the Influenza.* By Dr. H. SCHWEICH.—*Berlin*, 1836. 8vo. pp. 188.

2. *Die Influenza oder Grippe, nach den Quellen historisch-pathologisch dargestellt. Eine von der medicinischen Facultät zu Berlin gekrönte Preisschrift.* Von Dr. GOTTLIEB GLUGE.—*Minden*, 1837.

*An historical and pathological Treatise on Influenza; being an Essay rewarded by a Prize by the Medical Faculty of Berlin.* By Dr. GOTTLIEB GLUGE.—*Minden*, 1837. pp. 167.

3. *A Treatise on the Influenza of* 1837; *containing an Analysis of one hundred Cases observed at Birmingham, between the* 1*st of January and the* 15*th of February.* By PEYTON BLAKISTON, M.D., Med. Lic. Cantab.—*London*, 1837. 8vo. pp. 60.

4. *Report upon the Influenza, or Epidemic Catarrh of the Winter* 1836-7. By ROBERT J. N. STREETEN, M.D. (*Transactions of the Provincial Medical Association*, Vol. VI.)—*London*, 1838. 8vo. pp. 67.

THE present period, when the attention of every medical practitioner in the kingdom must have been called to the subject of influenza, during the prevalence of an epidemic as general at least as ever was experienced in these islands, seems favorable for introducing to the notice of our readers an account of the history of this interesting disease. In order that we may give the abridgment which we propose of the narratives of Drs. Schweich and Gluge, unbroken by our own commentaries, we shall previously introduce a brief notice of the epidemic with which we have been so lately visited; chiefly that the reader may be able, whilst perusing the histories of the German authors, to compare the different epidemics with that of 1837, as well as with those of 1830-33, described especially by Dr. Schweich. In addition to the information derived from our own observation of the late epidemic, we shall again refer to the pamphlet of Dr. Blakiston, to which we made a short allusion in a previous Number, and to the article in the Transactions of the Provincial Association. The former treats simply of *facts* which have fallen under the author's own notice, and conclusions apparently justified by these. It leaves untouched the history of previous epidemics, and the peculiarities (if any) of season in which the disease of 1837, or any previous epidemic, occurred. Like all the other works, it contains nothing satisfactory on the pathology of the disease. For the consideration of the epidemic of 1837, the arrangement adopted in the Transactions is that which we shall follow. The materials of the paper contained therein consist of an analysis of replies, made by practitioners from all parts of the country, to a list of queries sent to them by the council of the Association. The information which has been thus collected is of considerable value, and is most ably reported by Dr. Streeten: but, in any future application of a similar kind which may be made, it will be desirable, as far as possible, to define the subject of enquiry. The reporter remarks on the uncertainty of some of the returns, from a want of such definition; to avoid which it would have been preferable to obtain a description of the symptoms and other attendant circumstances which were regarded by the respondent as constituting influenza, and as distinguishing it from ordinary catarrh, or from any other disease to which it may be nearly allied. A circumstance of another kind must also be taken into consideration, in estimating the value of collations of this description; and that is, that the replies are made by practitioners whose employment is among very different classes of society; and it would be an invidious (although a necessary) question, Is your experience chiefly among the poor or among the rich? And, unless the replies are obtained from all the practitioners in any one place,—such, for instance, as a large town,—the mortality and some other circumstances cannot be well estimated. Again, although it is to be inferred that the majority of the replies are made from personal knowledge, still what is the limit between those thence derived and those which are from hearsay alone? How is a mind, not unfairly sceptical, to rest satisfied with the sort of evidence which is contained in "It would be difficult to point out a person who had not the disease;" and is one hundredth or one thousandth of the material of that statement the result of personal experience? This is certainly selecting an extreme case, but it is not an unfair illustration.

Circumstances of this kind, together with others which will strike any one reading reports similar to that in the Transactions, should be attended to, and made allowance for: but, with all this allowance, the report is very valuable, and we shall now proceed to its analysis.

Concerning *the time at which the influenza appeared*, the replies vary from the third week in November to the last week in January; but it is generally agreed that the period of its greatest prevalence was from the middle of January to the end of the first week in February. The accompanying table renders it evident that no conclusion can be drawn from the accounts which have been communicated to the Association, as to there having been any regular progression of the disease from one part of the kingdom to another.

| DISTRICT. | COMMENCEMENT. | TERMINATION. |
|---|---|---|
| Northern | November; middle of January. | February 8; April. |
| Midland | November; January 16. | Middle of February; May 1. |
| Western | End of December; February 2. | February; beginning of April. |
| Southern | Middle December; midd. January. | Middle of February; May. |
| Eastern | End December; beginning January. | Beginning February; midd. March. |

It will thus be seen that an extended range has been given to the whole epidemic; owing, probably, to its reappearance in the months of March and April.

An affirmative reply is almost universal to the query, *Did it attack a great many individuals at the same time?* Some, however, as Dr. Shapter at Exeter, and Mr. Maul at Southampton, refer to sporadic cases preceding the general attack.

The opinions are not uniform as to the *age, sex, and temperament particularly attacked by the disease.* More than half the replies, it is stated, speak of the indiscriminate character of its attack; but some, although in various degrees, except young children from liability to the disease. Mr. Prichard, of Leamington, gives the following statement of the ages in 170 cases occurring in his practice.

| | | |
|---|---|---|
| Under 14 years . . . | 26 cases; | about one sixth. |
| Between 14 and 65 . . | 119 | 144; about five sixths. |
| Above 65 . . . . . . | 25 | |

The Chichester Report, which contains the result of the experience of all the medical men in the city, says, "In regard to age, it seems to have almost equally attacked young and old. Of cases recorded, the greater number appears to be at the periods under ten and from thirty to forty; but the difference in the intermediate decades was trifling, and the uniformly decreasing numbers beyond forty would probably about tally with the small population of those ages." The following is a table of Dr. Blakiston's cases, relating as well to sex as to age.

| | Males. | Females. | Total. |
|---|---|---|---|
| Between 1 and 5 years . . | 2 | 1 | 3 |
| .. 5 and 10 . . . . | 2 | 0 | 2 |
| .. 10 and 20 . . . . | 5 | 7 | 12 |
| .. 20 and 30 . . . . | 8 | 15 | 23 |
| .. 30 and 40 . . . . | 13 | 8 | 21 |
| .. 40 and 50 . . . . | 6 | 13 | 19 |
| .. 50 and 60 . . . . | 9 | 3 | 12 |
| .. 60 and 70 . . . . | 3 | 4 | 7 |
| .. 70 and 80 . . . . | 0 | 0 | 0 |
| .. 80 and 90 . . . . | 1 | 0 | 1 |
| Total . . . | 49 | 51 | 100 |

Sex and temperament do not appear to have influenced the liability to attacks of influenza: but, whatever may be the fact as to the liability of children to the disease, three fourths of the accounts speak of its having been milder in childhood than at more advanced periods of life; although the age of childhood was not without its severe attacks. In our own practice, several children died of pneumonia produced by influenza; but they were greatly predisposed to the disease, and previously out of health. That *the aged suffered the most from the disease* is an almost universal opinion. In reply to the query, *What age suffered the most?* Dr. Hastings says,

"From sixty upwards. I answer this question most unhesitatingly. Under the age of sixty, persons, male as well as female, required, many of them, but slight attention to get safely, and in a few days, through the malady; but all of those, indiscriminately, male and female, who were so far advanced as sixty, suffered most severely, and had a long and dangerous illness, being confined to bed with cough and copious expectoration for some time. Of twelve persons above the age of sixty attacked, all were in bed for a week, all suffered most severely from profuse muco-purulent expectoration, all became considerably emaciated; eight were in bed for a fortnight and had a dry tongue, with small feeble pulse; four were in bed for a month, all the time so critically ill that I scarcely expected them to live from day to day; and two died within nine days of the attack. The four old persons who were in bed a month, have not yet [July?] quite recovered, and neither of them left the house till the month of June. Among the persons attacked below sixty, although in number thirteen times more than those above that age, I had comparatively few that were in bed a week; and those were persons who had been previously ill, either with pulmonary or other complaints." (p. 513.)

The testimony is general as to the *extensive diffusion of the disease.*

The replies to the question of the *proportion of deaths to the numbers attacked* are very indefinite. As far as an average can be made, it would appear to be about 2.3 per cent. On this subject we must refer to what we have already said of the great uncertainty of any attempts at very accurate numerical proportions. Next to *old age*, and in Dr. Blakiston's practice, the influenza "only proved fatal in those cases where the persons whom it attacked had been enfeebled by old age or chronic disease." Disease of the pulmonary organs (especially bronchitis and asthma) is mentioned as most frequently predisposing to a fatal termination of the disease. Dr. Shapter observes, that "the circumstances which particularly predisposed to a fatal termination were, amongst children, hooping-cough, and the recently having had some of the infantile eruptive diseases, which prevailed very much during the preceding November and December: amongst the more advanced in life, pectoral weaknesses generally, but more especially asthma:" and the comparative frequency of such affections in old age probably explains the greater fatality of the disease at that period. Dr. Brown remarks, "Besides the time of life, old age, and infancy already mentioned, chronic thoracic disease, or peculiar proneness to such disease, predisposed the patients to a fatal termination. Of the aged persons who died, in almost all there was some previously existing disease, generally chronic bronchitis, affection of the heart, or both conjointly." It appears that free livers were in some instances rapidly carried off.

*The duration of the disease* appears to have been very uncertain. Dr. Streeten, from a careful consideration of the replies, thinks that the disease may be divided into an acute stage, lasting generally from two to four or five days, the disease frequently terminating altogether at the end of that period; and a second, or more chronic stage, in which the symptoms continued in a slighter form for a period varying from five to ten days, or even a fortnight more: and many, after this, were affected with a state of general debility for an indefinite period. *Relapses* are said by some to have been frequent; by others, the opposite is stated; and this discrepancy attends accounts from the same locality,—e. g. Liverpool.

It does not appear that *individuals exposed to changes of weather in the open air were more liable to the disease than those confined chiefly to the house.* Some assert that the former appeared less liable; and the exceptions to this statement are few. From our own experience, we should say that, although, in innumerable instances, persons shut up in their houses suffered from the disease, yet we think that persons exposed to the ordinary exciting causes of catarrh were by far the most liable. For this reason, the servants in families were more frequently attacked than the members of families. Many individuals confined to their rooms escaped, whilst most of the other inmates of the house were attacked.

It is an almost universal opinion that the disease is not *contagious.* Mr. Maul states a fact, without however drawing any conclusion from it, (which is the only fact at all important on the opposite side,) that "if an individual come from a distance with the disease, the inhabitants of the house in which he arrived were usually attacked." The aggravation of pulmonary disease which existed at the time of an attack of influenza seems, with few exceptions, to have followed the disappearance of the latter; and this appears to be especially applicable to phthisis: and Dr. Hastings observes, "I may also remark that this is not confined to pectoral complaints. I find muco-gastritis and muco-enteritis of long standing referred in its commencement by patients to the influenza." Dr. Blakiston has detailed a case of phthisis, in which the disease did not appear to be at all worsened by the concurrence of the influenza. There is a statement also made by Mr. Welchman, of Kineton, to the same effect; but this and one or two similar statements can but be regarded as exceptions.

To the following queries the replies are doubtful, or none at all; indeed, there is no satisfactory evidence on the subject: *Were there any circumstances that appeared to exempt individuals from an attack of the disease? and, in particular, did the having been attacked during the last similar epidemic of the year* 1834 *appear to afford any protection?*

After a careful examination of the *symptoms* described as belonging to the disease by all the respondents, the reporters think that no very appreciable variation existed in its general features in the different localities. The symptoms are divided into febrile and those more immediately characteristic of the epidemic. The febrile symptoms, which it is needless to specify, sometimes commenced with actual rigor, the peculiar symptoms of the disorder showing themselves when the febrile state became fully developed: they were usually mild, less frequently more severe, sometimes typhoid. Very commonly the pains in the back and loins were more than belonged to the febrile state, and should rather be classed as symptoms of the epidemic. The catarrhal symptoms were very various in degree, from the mildest to the most violent: "the sense of weight and frontal headach were very prominently marked, being recorded in almost all the returns." Occasional lancinating pains over the eyebrows, of short duration, were among the early symptoms. The pain sometimes extended all over the head. There appears to have been a peculiarity in the violence of this headach. Soreness and rawness of the fauces were occasional, with "redness and tenderness" mentioned in the Chichester Report. Tightness and constriction of the chest were frequent, with more or less soreness beneath the sternum; but the symptom to which the greatest prominence is given is the cough, "which is variously described as being short and harassing,—troublesome and frequent,—preventing sleep,—very distressing, from its aggravating the pain in the head; sometimes as severe, violent, or coming on in frequent paroxysms of long duration. Some patients coughed little in the day, but much in the night; and in all cases, in some parts, there appeared to be a marked increase of cough, as of other symptoms, in the evening. Hæmoptysis is mentioned as having occurred with this cough to an alarming extent. "The expectoration is by no means so generally noticed; but, when it is mentioned, it is stated to have been scanty, difficult, and consisting of clear viscid mucus at the commencement, afterwards becoming more copious and free, opaque, and muco-purulent in its character, and occasionally tinged with blood. In some cases it is described as excessive and profuse." Among the aged patients who recovered there was often, for a time, a cough of a peculiar character, occurring in several in severe and prolonged paroxysms, terminating in scanty expectoration: in these cases there was no sonorous or constant mucous rhonchus.

The respiration was "in some cases short and hurried, or uneasy and oppressed, in others difficult. Pains in the chest are mentioned in some of the returns, in addition to the soreness under the sternum; and in one these pains are described as having been acute and lancinating. Examination by the stethoscope, according to Dr. Shapter, revealed the existence of sonorous and sibilous râles, and, for the most part, also a well-marked crepitation, in some part of the thorax, generally the lower portion." But Dr. Blakiston states that, "in seventy-five cases, no râle whatever was heard;" and on this point he gives the following table:

| | |
|---|---|
| No râles, in . . . . . | 75 cases. |
| Sibilous and sonorous râles alone, in | 5 |
| Sibilous, sonorous, and mucous, in . | 14 |
| Sibilous, sonorous, and subcrepitant, in | 2 |
| Mucous gurgling alone, in . . | 3 |
| Crepitant râle, in . . . | 1 |
| | 100 |

In many cases, even of moderate severity, there seemed a contradiction between the pectoral sound of the presence of sputa and its actual expectoration: whilst standing by the patient, one expected every minute to find the loose-sounding cough effect the discharge of the mucus; but none came.

The *circulation* was rather depressed than excited; the pulse, although quick, being small and feeble; and in only two instances being mentioned as full and soft.

The symptoms indicating derangement of the *digestive organs* were pain, tenderness, tension of the epigastrium and abdomen; anorexia; thirst, never very urgent; nausea; vomiting; furred tongue, sometimes becoming brown and dry, sometimes throughout the disease having looked as if covered with a layer of white paint. Dr. Barlow remarked that, when the chest-affection was trifling, the special irritation of the epidemic appeared to be seated in the stomach and bowels. The bowels were sometimes constipated, at others relaxed. Diarrhœa was occasionally observed; but the mucous membrane of the large intestine seemed to be much more rarely affected than those of the pharynx, trachea, and bronchi. In seventy of Dr. Blakiston's cases they were constipated. In Chichester, diarrhœa had prevailed before the occurrence of influenza. The urine was generally scanty and high-coloured; rarely abundant and limpid; sometimes becoming thick or whey-like, and depositing a copious sediment.

The symptoms ascribable to the *brain, spinal marrow, and nervous system* were great prostration, pains in the back and loins, and various neuralgic affections, sometimes very intense. These severe pains in the back, lumbar and sacral regions, were felt, in some places, in almost every case; the pain sometimes descending along the thighs and legs, sometimes shooting across under the scapulæ, and running along and around the arms. Violent pains in the eyeball and in the ears, or one ear, were among the neuralgic pains. Examples likewise occurred of severe spasms in the limbs, attended with a sinking pulse and Hippocratic face; and spasms of the muscles of the trunk were not uncommon in some parts. The peculiar prostration reminded one patient, who had had cholera in India, of what he suffered on recovering from it: indeed, it appears sometimes to have approached to the collapse of cholera. Dr. Davis, of Presteign, remarks, "that the *prostration of strength was instant and universal,* and attended with *extreme depression of spirits, and, in a vast majority of cases, spontaneous diarrhœa:*" and a peculiarity in this prostration was the length of time which the patient continued to labour under it, after the cessation of the other symptoms of the disease.

We have thus sketched the common symptoms of the epidemic, which were liable to varieties of intensity and combination, as is the case with other epidemic diseases. Certain *unusual symptoms* occurred in the practice of different individuals. Dr. Brown mentions the occurrence of three fatal cases of meningitis, in adults; and Dr. Hastings likewise mentions one such case which recovered. Acute pain in the head, relieved, after a week's duration, by a discharge of pus from the ear; abscesses of the ear; delirium; apoplexy; sudden insensibility of one or two hours' duration; convulsive attacks; syncope and intermitting pulse; excessive pains in the abdomen, usually midway between the umbilicus and symphysis pubis, sometimes with constipation, sometimes with mucous and sanguinolent stools; pain in the pubic region, and retention of urine; various neuralgic pains; soreness of the lips, mouth, and fauces, of unusual severity ;—are among the less frequent symptoms which accompanied the epidemic in different parts.

The experience of medical men with regard to the *Treatment of the Influenza* of 1837 cannot be regarded as very satisfactory. The main characteristic of the treatment mentioned in the replies appears to have been the cautious employment of evacuants generally; the use of diaphoretics and mild aperients in the first stage, with diluents, regulated temperature, restricted diet, and a cessation from all active pursuits. In severe cases, occasional local bleedings and counter-irritants to the throat or chest, with confinement to bed, were added. In the second stage, expectorant and anodyne medicines, with sulphate of quinine or mild tonics where there was much debility, are most commonly recommended. In the relapses, a more active treatment, with the freer use of evacuant remedies, (especially when inflammation attacked any organ,) was employed. General bleeding appears to have been very rarely employed, and is often spoken of as injurious. The almost unanimous statement is, that venesection was not employed except in acute inflammation of the pulmonary organs. In these cases our own experience is in favour of bleeding: the blood which was drawn presented the usual buffy coat. The Chichester Report, when speaking of the evil effects of general bleeding, says, "It is right to state that a very small quantity of blood, drawn from the schneiderian membrane, relieved the distressing headach in a marked manner: even a few drops accidentally flowing in two cases gave marked relief." Local bleeding is more recommended than general, though rarely to any extent. Various counter-irritants are well spoken of, especially in old people, to the chest and other parts requiring their use. Emetics are recommended by some practitioners, early in the disease. On this subject Dr. Blakiston says, "When the tongue was covered with a thick, soft fur, and when much nausea and vomiting were present, a brisk emetic produced a great amendment." Various expectorants and anodynes were used. Opinions varied as to the propriety of the latter. On this point Dr. Barlow says, "Some practitioners withheld opium and had protracted disease, as I had occasion to witness. There being no counter-indications, I combined it throughout, and with decided advantage." The exhibition of tonics and stimulants is also a point of practice on which some diversity of opinion existed. Much mischief appears to have arisen from their indiscriminate use in an early stage of the disease, in accordance with recommendations of the public press. Quinine is said by some to have been useful during the debility which attended convalescence. Mr. Myles says, that in old people, when the cough was a prominent symptom, he found a blister to the chest, with the sulphate of quinine internally, to act as a specific in all the cases he attended. Mercury and antimony were used, and not only as cathartics and diaphoretics, either alone or combined. Dr. Baird's practice was to administer three grains of calomel, with one grain of tartarized antimony, twice, thrice, or even four times in the twenty-four hours. "The effect of this powder," he says, "was to produce extreme nausea for the space of an hour, and frequent vomiting; to cause a vast discharge of purulent-looking matter from the lungs, excite a copious diaphoresis, and procure several dark pitchy evacuations from the bowels. So soon as the mouth became slightly affected by the calomel, (and in many instances before this was apparent,) the cough and expectoration had been greatly diminished, the restlessness had ceased, the countenance and eyes had assumed a more natural expression, the tongue had begun to clean at the edges, and the pulse restored to its natural state." A light, diluent, or farinaceous diet is generally recommended; though, from some of the returns, one rather more nutritious seems to have been occasionally found necessary. But the diet, like the treatment, was properly regulated by the symptoms in individual instances; not (as was too often the case with both) according to preconceived notions of the nature of the epidemic. In strictly inflammatory cases, the antiphlogistic regimen was requisite in all its forms; but, in the more general form of the malady, frequent mild nutriment, where it could be taken, was very beneficial. Much mischief was done by routine practitioners, haunted by fear of debility and typhus, enforcing a diet of animal food and strong drinks. Certain communications in newspapers and medical journals must have led to very injurious consequences in this respect. Dr. Blakiston speaks with commendation of the etherial tincture of lobelia in the states of congestion or inflammation of the bronchial mucous membrane attending the disease. According to our own experience, the simple saline medicine, or the liquor ammoniæ acetatis diluted with water, with a few drops of antimonial wine, were as generally beneficial, in ordinary cases, as any thing.

We pass over, for the present, the various accounts of atmospheric phenomena, and some interesting meteorological observations by Mr. William Addison, contenting ourselves with the conclusion at which he has arrived, "that it must be evident that the exciting cause of influenza cannot be found in sudden vicissitudes of temperature, great heat or cold, damp weather or melting snow; however much all or any of these circumstances may predispose to or originate the more ordinary catarrhs, eruptive fevers, and other disorders of spring, autumn, and winter." Dr. Gluge has on this point alluded to a remark of Sydenham, "that, after a sufficient observation of the weather during many years, he found every kind of weather accompany every kind of epidemic;" and Dr. G. adds, that he "has arrived at the same result after having examined the various authors who have mentioned the condition of the weather in the various epidemics of influenza." Dr. Schweich has endeavoured (with what success we shall hereafter see) to attribute the occurrence of influenza to other causes.

Before we notice the history of the disease contained in the works of the German authors, we will abridge a notice made by Dr. Gluge, at the end of his volume, on a pathological appearance said to have been observed in the fatal cases of pneumonia connected with influenza. Dr. Gluge states that he has seen the appearance himself. He alludes, as a standard of comparison, to the false membrane of croup, and to similar exudations which have taken place in the bronchial tubes; and says that in the above cases a similar appearance existed in the lungs. In all these cases there are found, in the hepatized portions of the lung, white, elastic, firm cylinders, occupying the bronchia, which may be sometimes followed from the fourth or fifth subdivisions of these tubes, into such as have not more than one quarter of a line of diameter. By some care, Dr. Gluge could detach this substance, resembling in arrangement the bronchial ramifications. The inner membrane of the bronchia is extremely reddened, but not softened; and the formation of these cylinders, as well as the redness of the bronchia, is absent in the healthy parts of the same lung. Dr. Gluge has never met with a similar appearance in hepatized lung unassociated with influenza; but he merely mentions the above as a fact, without connecting any hypothesis with it.

Attempts have been made to date the existence of influenza at a very remote period. Dr. Most has extracted from the writings of Hippocrates an account of a disease which he regarded as influenza, but which differed from it in several important particulars. Some epidemic catarrhs which prevailed in the fourteenth and fifteenth centuries are recorded, but these can scarcely be regarded as influenza, of the existence of which we have no credible accounts previous to the tenth century. Such is the opinion of Dr. Schweich; and the instances brought forward by Dr. Gluge of the earlier existence of this epidemic are not satisfactory. Since the sixteenth century it has frequently recurred, and the extent of country over which it has passed, as well as the large number of individuals whom it has affected, has afforded ample opportunity for local descriptions of the disease, of which medical authors have considerably availed themselves. It is from these sources that Drs. Schweich and Gluge have collected the materials for their essays.

The plan which the former has adopted has been, after some general considerations of the subject, to describe the influenza as it occurred in 1831-33; and then to notice, in their regular series, its previous attacks; comparing the characteristics which distinguish each with the epidemic which was first of all described. Dr. Gluge has commenced his essay by a short critique on the writings of various authors on influenza, in which he finds fault with Dr. Schweich, partly for incorrectness, partly for not having extracted as much valuable information as he might have obtained from English authors, and partly for certain fancies in which Dr. Schweich has indulged himself and amused his readers, respecting the causes of influenza, to which we shall allude hereafter. After a general description of the disease, a consideration of the causes to which it has been ascribed, its geographical extension, he also considers in detail all the instances of epidemics of influenza which are on record. He appears to have omitted the examination of no work in which information might be obtained: at least, if we may judge from the annexed Bibliography of Influenza.

We shall follow the arrangement of Dr. Schweich in what follows, which will be solely analytical.

Our authors are at issue respecting the course which has been taken by epidemics of influenza. Dr. Schweich says that this course has varied according to the situations in which the disease originated; that this origin has been most frequently in the north, whence the disease has extended in a south-westerly direction; that it has less frequently passed from south to north, or from north-east to north-west, and never from west to east or from north to south. One instance of the disease is recorded which commenced in various places, and followed different directions.

Dr. Gluge on this subject says, that we observe, in the history of influenza, until the end of the sixteenth century, that its course was always from west to east; but that, since the sixteenth century, all the epidemics have taken the opposite direction, from east to west. If the above statement is to be understood literally, Dr. Gluge is certainly in error, especially with respect to the former part; for the epidemics previous to the sixteenth century, as quoted by Dr. Gluge, are no evidence of progression from west to east; a direction which, we are disposed to think, with Dr. Schweich, is never taken by influenza. Thus, in Dr. Gluge's own table, it is evident that he knows nothing of the course of the epidemic of 1387, although he has quoted it as passing from west to east; the only place at which he mentions that it occurred being Florence. And again, with regard to the epidemic of 1557: Sicily, Nismes, Holland, the places at which the disease is mentioned as having progressively appeared, cannot be instanced as any proof of progression from west to east. Similar objections apply to the epidemics of 1580 and 1593. Indeed, it is difficult to see what motive Dr. Gluge could have had for making so very absurd a table. We are, on the whole, disposed to think that Dr. Schweich is correct in the directions which he has ascribed to the epidemic; although, of the diseases happening at an early period, the proof is very unsatisfactory indeed.

The different epidemics, says Dr. Schweich, have always been preceded, accompanied, or followed by certain cosmic phenomena,—such as comets, northern lights meteoric stones, peculiar fogs, earthquakes, volcanic eruptions, floods, remarkable changes in the weather, and by generally disturbed vegetation, and contagious diseases among men and animals. As a general law, in which Dr. Gluge does not agree, any more than he does with the foregoing assertion, Dr. Schweich states that the extent of every epidemic of influenza has an inverse ratio to its intensity. Where few individuals are affected, those few suffer more from the disease; when its occurrence is sudden and general, it is very mild. Between the years 1830 and 1834, the influenza twice traversed Europe. The latter epidemic attracted the notice of medical writers more than the former, both on account of its speedy recurrence and because the cholera, which had diverted their attention from the previous epidemic, had then ceased. The chief difference between the two epidemics consisted in the greater intensity of the disease of 1830-32, compared with the greater extent of its successor. In Europe, the former commenced in the east; but it had previously existed in Australia. It appears probable that the two epidemics of this year had an independent existence, and followed a course irrespective of each other. In the northern hemisphere, it was first noticed at Moscow; whence, in eight months, it extended to St. Petersburgh, Warsaw, Frankfort, Paris, London: three months subsequently it appeared in Italy, and shortly afterwards in Gibraltar. It passed with equal rapidity to America, which it reached in 1832, about fifteen months after its commencement. It recurred in 1833, originated in the north-east, and passed over Europe. It is uncertain whether the previous disease had traversed America, passed round the globe, and reappeared in Russia; then giving rise to the appearance of a second epidemic. There was but a few days' interval between its appearance in St. Petersburgh, Moscow, Odessa, Alexandria, and Paris. Gluge describes them as two distinct epidemics; quoting Radius as an authority for the latter "having appeared in a very different form, the catarrhal symptoms having often been inconsiderable; the symptoms frequently consisting only in faintness or trifling rigor, with feverish heat, disposition to perspiration, a heavy pain over the eyes, nausea, and diarrhœa; but that prostration was always present." These symptoms cannot be regarded as constituting any material difference, particularly as Dr. Gluge does not say on what extent of observation the above remarks are founded.

Among the numerous terms which have been applied to the disease, Dr. Schweich prefers either "epidemic nervous catarrh, or nervous catarrhal fever;" the essential characteristics of the epidemic being nervous and catarrhal, which are more or less evident in its different stages. He describes the disease as having consisted of four stages: 1, premonitory, or of derangement of general sensation; 2, stage of increase, or of nervous congestion; 3, stage of decrease, or of general effort at secretion; 4, of universal loss of power, with commencing convalescence or approaching death. Neither in its separate stages nor in the disease as a whole was there any definite duration. The signs of the first stage were those of derangement of general sensation, connected with universal debility. In addition to these, in the second stage, there occurred transitory aching pains in various parts of the body, with catarrhal congestions in most of the mucous membranes; sometimes of greater violence in those of the chest than in those of the abdomen. These were distinguished by their usual symptoms. In the cases of children, observed by Dr. Schweich, there was an itching sensation in the larynx, accompanied in many instances with croupy respiration. The skin was always dry, its temperature commonly elevated, but occasionally depressed. A remittent fever almost always accompanied the disease, with evening exacerbations. This was sometimes very slight. The expression of the face was that of intoxication. The disease manifested itself in three principal forms: 1, a nervous catarrhal; 2, a nervous synochal; 3, a simply nervous: the last being by far the most frequent, the second the most uncommon. In the first the pains were slight, the mucous membranes much affected, and the character of the fever torpid. In the second the symptoms were more marked; the pains considerable, and accompanied with spasms; nausea and vomiting took place; there was much determination of blood to the head, and symptoms which induced a belief of the existence of inflammation, the treatment of which by bleeding produced injurious effects. The blood, when drawn, separated into a soft coagulum and turbid serum; faintness being readily produced. The weakness in this form was more than in the others. The third form was the slightest and most frequent: from its slightness its existence might be overlooked. It was marked by derangement of the general sensation; slight alteration of the face and voice; trifling cough; some anorexia, and other unimportant symptoms. The peculiarity of this form was, that it continued during eight or ten days; when, towards evening, a fever appeared, which, if well managed, put an end to the disease. The continuance of the second stage was, in the first form, very short; never extended beyond two days in the second, and was undefinable in the third. The third stage was marked by general efforts at secretion, and the secretions were evidently of a critical character; as was shown by the improvement of the symptoms. The skin became moist, and sometimes covered with sweat; sudamina and other cutaneous eruptions, such as scarlatina and measles, occurred; the parotid glands occasionally enlarged; free secretions occurred from the whole mucous membranes of the alimentary canal, the frontal sinuses, nostrils, and bronchia. Epistaxis likewise took place. The urine became more abundant; and, after a duration of three or four days, these symptoms, together with the fever, mostly disappeared. The fourth stage was marked by a continuance of debility after the previous symptoms had all ceased; and this continued for some time before convalescence was quite established. The course of the disease was modified by other circumstances. In the phlegmatic temperament it was more slow, in the inflammatory diathesis more acute, and in individual constitutions modified by idiosyncrasy. In children there were frequent congestions to various organs, in such a degree as to simulate inflammations. In the north, the organs of the chest were more affected, and the fever possessed more of the synochal character; in the south, the nervous symptoms predominated. The debility of old age was most universally an unfavorable circumstance. The early fatal terminations of influenza were the consequence of injury of the lungs, apoplexy, marasmus senilis; but death occurred at a subsequent period in consequence of the development of phthisis, an effect of the disease. In some places—e. g. Berlin—the mortality, during the influenza, and for some time afterwards, exceeded the number of births. Those who suffered from chronic pulmonary complaints, and who were weakened by age, were cut off in the greatest numbers. Several instances of abortion and of premature delivery are recorded as having been produced by it.

Under the use of diaphoretics and a regulated diet, the disease was generally mild in its course, and sometimes terminated critically, without the development of the second stage. Emetics were very useful, when employed early. Antiphlogistics with narcotics relieved the pains and cough. Clysters were preferable to purgatives, given in the ordinary way. Purgatives and bleedings are almost universally admitted to have been injurious, although cases sometimes occurred which required their employment. The early use of stimulants was often found very prejudicial. The duration of the disease was from four to twenty days; but there was liability to relapse. The influenza, says Dr. Schweich, is distinguished from sporadic catarrh, as well as from the epidemic catarrhal fever which often occurs in spring and autumn, by the great debility, the disturbance of the general sensation, spasmodic pains, nausea, and vomiting, the great disposition to sweating and the occasional occurrence of exanthema; by its danger to old people, the peculiar expression of the face; but, above all, by its great epidemic extent. Its prognosis may be inferred from what has been already said.

Before we proceed to the early history of the disease, we shall extract from Dr. Gluge's work, his "description of the epidemic, generally, derived from the most trustworthy sources:"—Prostration and weariness to such a degree that the strongest constitutions are frequently rendered powerless; frequently, great sensibility to any chillings accompanying the whole disease; great discomfort, anxiety, and weariness of self and everybody besides; fixed tense pain of the head, mostly in the forehead and over the eyes, frequently increasing to giddiness; nights sleepless, either with delirium or lethargy. Together with these, have been observed disordered sensations, such as a sensation of coldness about the sagittal suture, and of a ball rising in the throat. The senses of sight and of taste were more often affected than the other senses. The organs of locomotion generally suffered: the muscles of the head, neck, back, and shoulders being painfully affected, the sensations in the extremities being sometimes as if the limbs were broken or dislocated. The thorax felt as if contracted and confined by an iron band. Anxiety and pain in the præcordia and stitch in the lumbar regions were complained of. When fever existed, it was commonly mild, with evening exacerbation. The pulse seldom rose to 120, was soft, small, always weak but seldom hard, when the fever was attended with inflammatory phenomena. Breathing was mostly short, anxious, sometimes attended with noises from accumulated mucus in the trachea. Bronchitis and pneumonia occurred. The nostrils were stopped up, and from them escaped a corroding fluid, sometimes mixed with blood, and producing relief. The face was swollen, often red as in erysipelas; eyes injected and filled with tears: the parotid and other glands were not unfrequently swelled. Hoarseness or loss of voice frequent. The inflammatory state of the respiratory organs produced a troublesome cough, greatly increasing the headach, accompanied with a thick, yellow expectoration; and this, most troublesome at night, was the most obstinate phenomenon of the disease. Sores occurred at the corners of the mouth: the tongue was covered, as it were, with white or yellowish cream. Thirst was rarely considerable. Anorexia, a desire for acids, eructations and vomiting, diarrhœa, were other symptoms: but constipation was more frequent than diarrhœa. The urine was clear, watery, or reddish, frequently turbid, sometimes having a sour smell, rarely normal, sometimes having a sediment, at others being only a little cloudy. The skin was dry, (sometimes having spots of purpura,) but after the first access of fever, sweat appeared on the whole surface, sometimes very excessive, and of days' duration.

If we look at the disease, says Dr. Gluge, as an epidemic, its characteristics are: the suddenness of its appearance in countries and among individuals; its regular advance; the fact of thousands being simultaneously attacked by it, the rapid development of its symptoms, the facility of relapse, and the obstinate and disproportionate debility which attend it.

Dr. Gluge has mentioned epidemics of influenza, in the years 1323, 1327, 1337, 1403, 1411, 1414, 1427; whilst Dr. Schweich says, that the earliest epidemic, which can properly be characterized as influenza, occurred in the year 1510: and certainly, if the definition of influenza is to be taken from either of these authors, there is in Dr. Gluge's book but very scanty proof of any epidemic of the disease prior to 1510. The epidemic of this year originated in the East and extended to Italy, France, and Spain. The righteous zeal of the court of Pope Gregory XIII. attributed its occurrence in France to a divine retribution, accorded to Louis XII. for his resistance to the power which so liberally "dealt damnation round the land;" but we are not told of the delinquency which subjected his holiness to a similar visitation. The disease was distinguished from that of 1830-34 by constant inflammatory fever, delirium, and subsultus tendinum, and it was very fatal from bad treatment. In Italy it was termed *coquelucha;* in France, *cephalite* and *coqueluche.* Early in the spring of 1557, measles, small-pox, and purpura prevailed among the children of Padua. During their continuance, in the following September, an epidemic cough, with violent pains in the head, suddenly occurred. It is described by Most, as having extended over the whole globe. Its general character was mild: but it was extremely fatal in Holland. Riverius states that a fetid sweat was a critical sign. Forest, speaking of this influenza, first mentions its influence in producing abortion "so that, within eight days, sixteen women died." Pains in the loins were so frequent, that few could walk.

The epidemic of 1580 originated in Africa, taking thence a northwesterly course, and branching off, as universally happened, in various directions; it eventually extended over the whole of Europe. The fever frequently assumed a putrid form, and the disease has consequently been described by some, rather as a putrid fever, than as a catarrh. Bleeding slayed its thousands, although some persisted in its unconditional recommendation. Its origin, course, and the putrid character of its fever chiefly distinguish it from the epidemics of 1830-34. Dr. Gluge gives an account of two somewhat doubtful epidemics of influenza in 1593 and 1626, to which Dr. Schweich does not allude. The influenza of 1658 was limited to a small part of England, and has been scarcely mentioned, except by Willis, as it occurred in Oxford and its vicinity. Dr. Gluge thinks that Dr. Schweich is wrong in thus limiting it; and quotes for this opinion a letter of Timæus, in which the disease is compared to its predecessor in 1580. Under entirely different conditions of weather, an influenza appeared in Germany and Switzerland, which showed itself in Hungary and France in September, 1675; it did not appear in England, until nine months after its commencement in Germany. Its distinctions were, very acute affections of the chest,—pleurisy and pneumonia being in England its frequent accompaniment; the abdominal organ remained unaffected, and there was no perceptible sweating.

There was a catarrhal epidemic (not alluded to by Dr. Schweich,) in 1693, which existed in England, France, and Holland.

Differing in many circumstances from that of 1830-34 was the influeaza of 1708-9. It was preceded by plague in many parts of Europe, and appeared first in Italy. Its course was from south to north. It was very violent and proportionally limited in its extent; the abdomen was rarely affected, and the crises were, not through the mucous membranes, but through the skin, manifested by acute cutaneous eruptions. In Italy, jaundice was critical. Dr. Gluge does not think that this was an attack of influenza, but he does not say what it was; certainly an unusual class of symptoms attended the disease.

An epidemic of influenza, extending from the north to the south-west, occurred in 1712. It varied from the disease of 1830-34 chiefly in a minor geographical extent, although great numbers were affected by it, in those places where it existed. In 1729-30, the influenza occurred with most unusual violence, and its fever was so inflammatory that Hoffmann termed it "febris synocho-catarrhalis." It probably originated in Russia, passed through the whole of Europe, and visited America. It appears to have spared nobody; but its fatality has been differently described by different authors. In London it was exceedingly fatal, and its character was the worst in England, Germany, France, and Spain. The unfavorable terminations of the disease were phthisis, hydrothorax, and anasarca. Its chief distinctions appear to have been, its inflammatory character, and the tendency to the formation of exanthemata. The disease was sometimes followed by a continued fever; and a double tertian occasionally occurred where the bilious symptoms were manifested. So many were ill of it in Moscow, that, says Dr. Gluge, "there were twenty patients in many houses; in Rome, there were 60,000 affected with it, and eighty physicians; in Milan 50,000; in Vienna 60,000; in London scarcely 100 remained free; and in Paris there were not priests enough unaffected to conduct the services of the church." We quote this, not because we have the least faith in the details, but because they are circumstantial evidence of the extent of the disease.

The influenza of 1731-35 (described by Gluge as of 1732-33) was preceded in England by a similar disease among horses. It broke out in Connecticut, and extended thence very rapidly to Massachusets and Newfoundland. About the middle of November, 1731, it appeared in Russia and Saxony, but no accounts exist of it from that time to the summer of 1732, when it is again spoken of as existing in Germany. The first place in which it appeared in Britain was Edinburgh, during September of the same year. In January, 1733, it appeared in Belgium, Italy, Paris, and Switzerland. In February, it wandered through Scotland and Spain, and by the end of the month, had spread all over

England and Ireland. It passed over to North America and took a southerly course as far as Mexico and Peru. The disease was distinguished by some peculiarities. Cynanche, pleurisy, and pneumonia, were frequent in Italy, and the symptoms of weakness were relieved by a spontaneous bilious diarrhœa. Extremely violent pain in the head was the precursor of death. The disease most usually terminated by secretions which occurred on the third, fifth, seventh, or fourteenth day. There was expectoration of fluid blood, followed by bad consequences. Asthma and dropsy sometimes succeeded it in females. There were occasionally swellings of the parotids, gums, and testicles. Ulcerated throat occurred in Belgium. The gastric affections were inconsiderable. Cutaneous eruptions and inflammation of the lungs were noticed in Germany, and a state of stupor, with subsultus tendinum and convulsions, sometimes preceded death. The appearance of cutaneous eruptions, such as urticaria, miliaria, and petechiæ, seemed in no way to increase the danger of the disease. The blood which was drawn is described as forming a thick, liver-like coagulum, from which no serum separated itself. In England and France, the disease was followed by an epidemic dysentery. Respecting its extent, Huxham says, "omnes pervasit domos, pauperum tabernas regumque turres; vix unus aut alter, rure vel in urbe, senex aut puer, robustus aut infirmus, evasit." The term influenza was first applied to the disease, which occurred in 1741-43; and which, in Messina and Sicily especially, was the precursor of plague. In some parts, it was preceded by a disease among cattle, attended with a bloody flux and eruptions on the skin. It commenced in Germany, and extended thence in a double direction; one, southerly; the other, north-westerly. It did not arrive in England until a year after its commencement. Its twofold course, and the great affection of the abdominal organs, chiefly distinguish it from the disease of 1830-34.

The influenza of 1756-58 and that of 1759 were so inconsiderable as not to require notice.

In October, 1761, an influenza commenced in Italy, arrived in Vienna during the following March, 1762, and in London in April. In the middle of the summer, it reached France, and in October, appeared in America. Its direction was from the east to Vienna, but from the north to France. The character of the disease in Vienna was the same as those of 1830-34. In general it terminated in recovery, but at Breslau, where it prevailed in February, eight hundred individuals fell a sacrifice to it. Dr. Schweich quotes Ozanan as his authority for this large and unusual mortality. Dr. Gluge says nothing about it, but considers Ozanan as a bad authority. The influenza of 1767 originated in upper Saxony, extended to England, and thence to France and Spain. At Eisenach it was preceded by a stitch in the side, from which few individuals remained free. Its extent was more limited than that of 1830-34, but its pathological character was very similar, excepting that in France there was a great tendency to a simple nervous fever. In 1775, an influenza commenced in Germany and affected the greater part of France. Though not more fatal, its character appears to have been more serious than usual. The chest and abdomen were both affected, and the crises proceeded from various sources. Among them were black and bilious stools. In France the disease was accompanied by the frequent formation of abscesses in the ears. Some had angina and dysentery; jaundice occurred in the predisposed. Many had a troublesome pricking of the skin with scarlet coloured spots, ulcers or red vesicles not at all critical: or a well developed erysipelas occurred. Fothergill says that in this epidemic dogs and horses were subject to the catarrh before men; and the same was remarked in France. In September, 1780, the ship Atlas, sailing from Malaga to Canton, was affected with a nervous catarrhal fever, which appeared at the same time on the shores of Canton and Coromandel. In 1781, an influenza travelled westerly through North America. In October, 1781, it reached Europe, when the intelligence was first given that it prevailed in the British army which was occupied in the siege of Negapotamia. Thence, it proceeded over Tartary and Siberia, appeared in Moscow in December, and in January, 1782, at St. Petersburgh, where 40,000 are said to have been affected by it. It passed through Prussia, Germany, Denmark; spread along the shores of the Baltic sea and through Belgium into England. At this time also, it traversed France and Italy, where it remained during the summer. It existed also in ships in the Atlantic Ocean. It then visited Spain and Portugal. Its course was from north-east to south-west. The chief differences between it and the epidemics of 1830-34, which, in its course, it exactly resembled, was the appproximation of its character to that of an epidemic bilious fever. The extreme and sudden depression of the nervous system was very striking in this epidemic. In some parts, there were very marked spasmodic attacks. In Leipzig the fever was intermittent. The glands of the head and neck were much affected in some cases. Bilious and inflammatory symptoms were strongly marked in Cassel. The disease in Lunemberg, more than elsewhere, seemed to confine itself to particular parts of the body. Some had stitch in the side, hæmoptysis, epistaxis, violent headach, and pains in the eyes; others, nausea, tension of the abdomen, and diarrhœa; others, inflamed parotids, abscess in the ears, eruptions upon the hands and feet, salivation, dysuria. Hemicrania was very frequent in Memel, accompanied with nocturnal delirium. In Königslutter, the disease was like typhus. Cutaneous eruptions were not uncommon in Italy, where the disease often assumed a putrid character. In most of the cases, the bilious character of the disease was more or less evident. Hepatitis, jaundice, bilious stools and urine, and bilious vomiting are the most frequent symptoms in all accounts. There appears, also, to have been a transition to tertian and quartan fevers; convalescence was very long and relapses frequent.

In 1788-91, a mild influenza, originating probably in Asia, and proceeding thence, passed through the whole of Europe, and then extended to America. In consequence probably of its mildness, the accounts which are recorded of it, are few. In England, a pain in the scrobiculus cordis, and greater affection of the head, appear to have been the chief distinctions between it and the disease of 1833-34. It is said by Rush, that children under eight years of age, were scarcely affected by the disease. He mentions, also, that so general was the coughing that the clergyman could not be heard in church. From the scarcity of the accounts, it may be suspected that the disease was very mild. In 1799, an influenza commenced in Russia and extended over the greater part of it, thence it traversed some portions of Germany and France, in which countries it took no determinate course. Italy was next affected. It appears to have spared the north of Germany, and not to have affected England, although it reached Scotland. It was distinguished from the disease of 1830-34 by the irregularity of its course, by its putrid, typhoid character in Germany, and by the addition, in the south, of acute inflammatory affections, as well as by the great frequency with which it appears to have affected hypochondriacal and hysterical individuals. Dr. Gluge speaks of hemorrhages from all the outlets, erysipelas, miliaria, swelled glands, delirium, as characterizing the epidemic; and that it left behind "a dread and depression of spirits as in those labouring under hypochondriasis, but which soon disappeared." With regard to this, also, it was observed, as some have remarked in other instances, the phthisical were not especially liable to be affected, nor did their disease become worse when the influenza left them.

In the foregoing account, we have made little allusion to the treatment which was employed in the various epidemics, as it would but have led to useless repetitions. Almost every class of medicines was at different times made use of, bleeding appears to have been most commonly condemned, although some have strongly recommended it; and, in other cases, the varying constitution of the epidemic was in part the cause of the difference of opinion on this subject. But bleeding was at times carried to a dangerous extreme; and in one attack of the epidemic in Russia, the mortality caused by bleeding was so great, that its discontinuance was ordered by an imperial ukase. The complications of inflammation or of very inflammatory fever appear to have been the only circumstances which justified bleeding, and even in these cases, its effects on the subsequent course of the disease were often manifestly injurious. Clysters or mild laxatives were found to be preferable to more active purgatives; emetics were often of considerable service. The indication, generally, seems rather to have been to promote a gentle action of the skin by mild diaphoretics, than to administer active sudorifics, by which inflammation was not unfrequently produced. In the early stage, stimulants and tonics were occasionally employed, and they have not wanted strong recommendation, but the time found to be best suited to their administration was the later period of the disease. In some epidemics, those of a milder character, a merely expectant treatment, consisting in the use of demulcents, constituted the chief remedial means. Bark, acids, camphor, opium, antimony, valerian, were employed by different practitioners; the reigning medical fashion, or the fancy of the individual, having probably a greater influence in determining their value, than the benefit which they effected on the disease.

The proximate cause of influenza, Dr. Schweich conceives to be an affection of the nervous system, extending from this to the vascular and muscular systems. This he conceives to be indicated by both the premonitory and other symptoms: the weakness, the subsequent fever, the altered blood, and the spasmodic pains in the muscles. True inflammation rarely exists; the blood never exhibits a truly inflammatory crust; false membranes are not formed; bleeding is almost always injurious, and the pains are not increased by pressure. Nervous congestions do occur, and inflammation is sometimes a consequence. In these cases there is an inflammatory crust on the blood, and bleeding is beneficial.

The spasmodic character of the disease is opposed to inflammation. The great debility manifests the affection of the nervous system, especially of the spinal marrow. The brain is seldom affected. From the alleviation which takes place from the various discharges in this disease, Dr. Schweich is disposed to regard them as critical. The influenza shows a great similarity to the febrile exanthemata, which always commence with catarrhal affections, and the occurrence of measles, scarlatina, and petechia, is not unfrequent in the disease. The character of the accompanying fever (which does not always exist) is influenced by the constitution of the individual and by the climate. In the north it is inflammatory; in the south torpid, and even typhoid. Old people appear to die of a nervous paralysis, and perhaps of the nervus vagus. In its various modes of appearance, influenza has shown a similarity to several epidemic diseases: to the English sweating sickness, by its mode of attack, great oppression, and sweating; and to the following diseases, by symptoms and characteristics which may be gathered from the preceding remarks,—to oriental cholera; to bilious, mucous, nervous, and typhoid fever; to scarlatina, miliaria, urticaria; and to petechial fever.

Considering the unsatisfactory nature of the occasional causes to which influenza has been ascribed, Dr. Schweich has produced an hypothesis of his own; one, it will be seen, which is nearly allied to the hypothesis applied by Dr.Buzorini to the explanation of the causes of typhoid diseases. He imagines that electricity being accumulated in the body, and being prevented radiating thence, gives the impulse to the disease. He adds, that if it can be shown that, during the prevalence of influenza, there is always an abnormal accumulation of electricity in the air, which, according to physical laws, is always an isolator of the electricity of the organism, this hypothesis should not be rejected until we possess something more satisfactory. The existence of miasma he conceives to be a purely gratuitous assumption. To attribute the disease to changes in the weather is unsatisfactory, as these changes often succeed the disease, and have often occurred without giving rise to it. Comets, meteoric stones, earthquakes, and volcanic eruptions have borne the blame of producing the influenza; and the previous objection applies to these, as also to the endeavour to explain its origin by the inspiration of microscopic animalcules. Dr. Schweich considers that, with respect to epidemic diseases generally, a more close investigation of electricity and of magnetism, during the existence of such diseases, may lead to useful results. All great changes in the atmosphere, and in terrestrial bodies, tend to alter very much the electric relations of the air; such as changes of weather in its temperature, moisture, volcanic phenomena, earthquakes, floods, &c. In this respect our author says, that the winds also require to be taken into account; as the north and east winds bring the positive, and the south and west the negative electricity. It is always set free by evaporation, and the northern lights appear to increase its quantity: but have such causes as these, which produce changes in the electric condition of the atmosphere only, always existed at the time of the appearance of the epidemics of influenza?

Dr. Schweich maintains that the result of an examination to this effect is favorable to his hypothesis. He then proceeds to show that, at the time when epidemics of influenza have prevailed, there have existed causes especially capable of changing the electrical conditions of the atmosphere. The chief among these are earthquakes, frequent and rapid alterations of the weather, from hot to cold, and moist to dry; rains and floods; offensive and thick fogs; northern lights; volcanic eruptions; whirlwinds; other conditions indicated by remarkable barometrical changes, &c. But, however coincident some of these may have been with certain epidemics, the conclusions which can be derived from them in support of the electric hypothesis appear to rest on a very feeble foundation. Six of the epidemics which are related in the previous history are without their accompaniments of such circumstances as are here said to change the electric relations of the air; and it requires no octogenarian experience to remember the occurrence of most of these circumstances without the production of influenza. The unsatisfactory character of the first steps of this hypothesis will excuse our following its author in search of the causes with which these terrestrial phenomena are associated. As the clue to its completion could not be found on earth, he has gone even beyond the lunar sphere in search of it, and has endeavoured to show that it exists in certain planetary conjunctions; the coincidence of which with some epidemics he has illustrated. In doing this we may perhaps commend his ingenuity, but there are few who, in the present state of our knowledge, and even with the assistance of Dr. Schweich's book, will not perfectly agree in his conclusion that "it cannot yet be determined by what planetary conjunction the influenza is occasioned." Dr. Gluge does not think the above conjecture worth the amount of notice which we have given to it. He is less prone to hypothesis, which he justly regards, when meddling with the above subjects, as rather premature, considering the amount of our present knowledge of the electricity of organized bodies.

Whether contagion is one among the means by which influenza is propagated, neither of our authors can decide. Dr. Gluge has said a good deal about it, but, with such a conclusion, the reasons can be but little acceptable.

For those who are desirous of possessing an elaborate history of influenza we strongly recommend the works of Drs. Schweich and Gluge, especially the former. To what precise degree either or both may be correct, can only be told by those who examine the many hundred volumes whence the two before us profess to be derived. We pretend not to have done this. There is, with the slight exception already alluded to, no morbid anatomy in either of the works. This, however, is a subject to be borne in mind by future observers of these epidemics.

